# Age and Sex Matter: Phenotypic Heterogeneity, Diagnostic Gaps, and Screening Tool Performance in Obstructive Sleep Apnea—A 10-Year Sleep Clinic Cohort Study

**DOI:** 10.3390/diagnostics16070983

**Published:** 2026-03-25

**Authors:** Asterios Tzinas, Aliki Karkala, Serafeim-Chrysovalantis Kotoulas, Georgios Kalamaras, Eleni Mouloudi, Eleni Massa, Athanasia Pataka

**Affiliations:** 1 Respiratory Failure Unit, G. Papanikolaou Hospital, Aristotle University of Thessaloniki, Papanikolaou Avenue, Exochi, 57010 Thessaloniki, Greece; kalamaras.giorgos@gmail.com (G.K.); patakath@yahoo.gr (A.P.); 2 Department of Pulmonology, Leuven University Center for Sleep and Wake Disorders (LUCS), University Hospitals Leuven Campus Gasthuisberg, Herestraat 49, 3000 Leuven, Belgium; aliki.karkala@uzleuven.be; 3 Adult ICU, “Ippokratio” General Hospital of Thessaloniki, Konstantinoupoleos 49, 54642 Thessaloniki, Greece; akiskotoulas@hotmail.com (S.-C.K.); elmoulou@yahoo.gr (E.M.); elenizioga@yahoo.com (E.M.)

**Keywords:** obstructive sleep apnea, gender differences, age differences, menopause, STOP-BANG, insomnia, cardiometabolic comorbidities

## Abstract

**Background**: Obstructive sleep apnea (OSA) is a highly prevalent and heterogeneous sleep disorder. This study aimed to investigate age- and sex-specific phenotypes of OSA in a large clinical cohort, with special emphasis on the effects of aging and menopause on disease severity, hypoxemic component, symptom expression, comorbidities, and screening tool performance. **Methods**: A retrospective cross-sectional analysis was conducted on 3736 patients with confirmed OSA referred to a tertiary sleep clinic in Greece between 2013 and 2023. Participants were stratified by age and sex. Women were further classified as pre- and post-menopausal using age as a proxy. Clinical characteristics, comorbidities, questionnaire data and polysomnographic indices were analyzed using parametric and non-parametric statistical methods. **Results**: OSA severity peaked in middle age and remained high in older adults, while nocturnal hypoxemia and cardiometabolic comorbidities worsened with age. Women presented at an older age and with higher body mass index but lower Apnea Hypopnea Index (AHI) compared to men, yet experienced significantly longer hypoxemia and more severe insomnia. Women over 45 years exhibited a markedly more severe and hypoxic phenotype and substantially higher rates of comorbidities. Screening tool performance varied across subgroups: STOP-BANG was the most consistent, while symptom-based tools performed poorly in young women and elderly patients. **Conclusions**: OSA is a dynamic dependent disorder with distinct phenotypes across the lifespan. Current screening strategies fail to adequately capture these variations, particularly in women and older adults. A personalized, sex- and age-specific approach to OSA diagnosis and management is essential to reduce underdiagnosis and improve outcomes.

## 1. Introduction

Obstructive Sleep Apnea (OSA) is characterized by recurrent episodes of partial or complete upper airway collapse during sleep, leading to intermittent airflow obstruction, oxygen desaturation, and sleep fragmentation. The core pathophysiological mechanisms involve a complex interplay between anatomical factors, such as a narrowed or collapsible upper airway, and non-anatomical as impaired muscle responsiveness, high loop gain (unstable ventilatory control) and a low arousal threshold [1].

Globally, OSA affects almost one billion adults and represents a major public health burden due to its high prevalence, chronic morbidity, mortality, and healthcare costs. The disorder significantly decreases quality of life and imposes substantial economic costs on healthcare systems worldwide [2]. The prevalence of OSA also increases with age [3]. At the clinically important Apnea Hypopnea Index (AHI) ≥ 15 level, OSA prevalence in the overall adult population ranges from 6% to 17%, whereas in the advanced age groups, this seems to be as high as 49% [3]. Young adults show an overall prevalence of around 16%, according to a 2024 meta-analysis [4]. Among middle-aged adults (30–70 years of age), approximately 13% of men and 6% of women have moderate-to-severe Sleep Disordered Breathing (SDB) (AHI ≥ 15), whereas 14% of men and 5% of women present AHI ≥ 5 plus symptoms of Excessive Daytime Sleepiness (EDS) [5].

Despite that OSA has been considered a male disease, females currently represent up to 40–50% of referrals at sleep clinics, even with ‘atypical’ symptoms like daytime fatigue, lack of energy, insomnia symptoms, morning headaches, mood disturbances and nightmares [6]. Men tend to have more severe OSA, with higher AHI, higher Oxygen Desaturation Index (ODI) scores and longer apneic episodes compared to women, typically reporting symptoms like snoring and EDS [7]. It is also significant to note that the prevalence of OSA in women increases after menopause, nearly reaching male levels, likely due to the loss of protective effects from female sex hormones, such as progesterone and estrogen [8]. The pathophysiology behind those gender differences lies in anatomy, upper airway dilator muscle activity, stability of the respiratory control system and hormonal effects. Men tend to gain weight more centrally than women, and this pattern probably results in men having more fat stored in upper airway structures than women [9]. Moreover, young healthy pre-menopausal women tend to have significantly higher peak phasic and tonic genioglossus electromyogram activity compared with men during wakefulness [10,11].

Given the clinical and polysomnographic heterogeneity of OSA across age and sex, there is a necessity for individualized diagnostic and treatment strategies. The aim of this study is to clarify the complex interactions among demographic (age, sex), clinical (insomnia, EDS), and polysomnographic (AHI, ODI, hypoxemia) factors that contribute to OSA susceptibility and heterogeneity.

## 2. Materials and Methods

This retrospective cross-sectional cohort study analyzed data from patients referred to the sleep clinic of the Respiratory Failure Unit at “Georgios Papanikolaou” General Hospital in Thessaloniki, Greece, between 2013 and 2023 for suspected OSA. During their initial visit, participants completed a series of standardized questionnaires, which included a comprehensive sleep habits questionnaire, the Epworth Sleepiness Scale (ESS), the STOP-BANG questionnaire, and the Athens Insomnia Scale (AIS). The cut-off points that were used for the screening tests were >10 for the ESS, ≥3 for the STOP-BANG and ≥6 for the AIS [12,13,14]. The diagnosis of OSA and its severity were confirmed through objective sleep testing, which involved either in-laboratory polysomnography (PSG) or Home Sleep Apnea Test (HSAT), following standard clinical protocols. Study participants were evaluated using either type 1 polysomnography (Nox A1 PSG System, Nox Medical, Reykjavik, Iceland) or a type 3 home sleep apnea testing device (Nox T3s HST, Nox Medical, Reykjavik, Iceland). Clinical data from 3736 patients with a confirmed OSA diagnosis were included. An apnea was defined as a ≥90% reduction in airflow lasting ≥10 s, while a hypopnea was defined according to the American Academy of Sleep Medicine (AASM) 2012 criteria as a ≥30% reduction in nasal pressure signal amplitude lasting ≥10 s, accompanied by a ≥3% oxygen desaturation or an arousal [15]. The AHI was calculated as the total number of apneas and hypopneas per hour of sleep. Patients were categorized according to OSA severity into three groups: mild (AHI 5–14.9), moderate (AHI 15–29.9), or severe (AHI ≥ 30). Patients with primary sleep disorders other than OSA (such as central sleep apnea syndromes, narcolepsy, or periodic limb movement disorder) as defined by the International Classification of Sleep Disorders, Third Edition (ICSD-3) [16], were excluded, along with those with missing critical data (such as AHI or key demographic variables).

**Figure 1:**
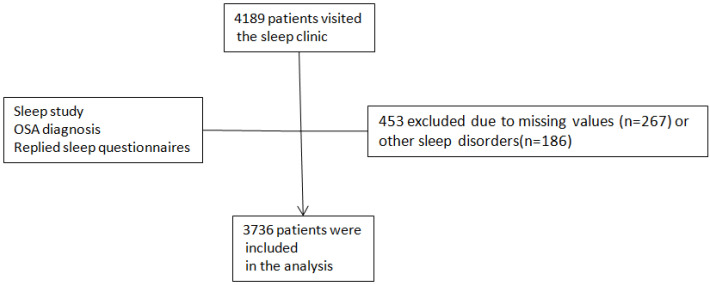
Flow diagram of patient selection.

Figure 1 illustrates a flow diagram showing the application of the inclusion and exclusion criteria:

The patient sample was stratified into three age groups: under 45 years, between 45 and 65 years, and over 65 years. However, for a more precise screening questionnaire validation across specific phases of adulthood, particularly capturing potential shifts in the elderly and the oldest-old subgroups, a more granular stratification according to the General WHO Age Categories (2025) was chosen, within the following strata: <45, 45–60, 60–75, and 75–90 years. Men and women were analyzed separately, treating sex not as a confounding variable to be adjusted for, but as a primary variable of interest and a key effect modifier. Furthermore, women were further stratified into two groups based on age: those aged 45 years and younger (a proxy for pre-menopausal status) and those over 45 years (indicative of post-menopausal status). The proposed cut-off age of 45 years for menopause is scientifically justified and consistent with established clinical and epidemiological definitions. Population data indicate that the typical age range for natural menopause is approximately 45 to 55 years. Therefore, using 45 years as a threshold provides an evidence-based and internationally consistent criterion that supports uniform classification, data comparability, and accurate clinical interpretation. We acknowledge that it does not capture individual variability in menopausal timing. Therefore, all interpretations regarding menopausal status should be considered approximate and hypothesis-generating rather than definitive. Smoking history, defined as pack-years, was obtained by patients’ self-reporting (number of cigarettes × years/20). Medical background related to comorbidities was obtained by self-reporting and verified by electronic medical records.

Data were analyzed using IBM SPSS Statistics version 21.0 (IBM Corporation, Armonk, NY, USA). The study followed the STROBE (Strengthening the Reporting of Observational Studies in Epidemiology) guidelines for reporting observational studies. Due to the large sample size (*n* = 3736), the principle of the Central Limit Theorem was invoked, supporting the use of parametric statistical analyses. Prior to analysis, the distributions of continuous variables were inspected both graphically and using normality tests. In cases of significant deviation from normality, robust non-parametric alternatives were applied. Descriptive statistics were used to summarize the data. Continuous variables are presented as Mean ± Standard Deviation, while categorical variables are reported as absolute numbers and percentages (*n*, %). Group comparisons for continuous variables across the three age groups were performed using One-Way Analysis of Variance (ANOVA). Non-parametric variables were analyzed using the Mann–Whitney U test or the Kruskal–Wallis test. In cases where the Kruskal–Wallis test showed statistical significance, post hoc comparisons were performed using the Bonferroni correction for parametric variables and the Mann–Whitney U test for non-parametric variables. Univariate and multivariate logistic regression analyses were carried out to calculate odds ratios (OR) with 95% confidence intervals (95% CI). Multiple regression analysis was performed in order to test whether the different questionnaire values remained independent after adjustment for BMI, gender, AHI, smoking burden, comorbidities (arterial hypertension, diabetes, stroke, arrhythmia, respiratory disease) and diagnostic modality.

The assumption of homogeneity of variances was assessed with Levene’s test, and Welch’s ANOVA with Games–Howell post hoc testing was applied where variances were unequal. For comparisons between two independent groups (e.g., men vs. women), independent samples *t*-tests were used. Categorical variables, such as prevalence of Arterial Hypertension (AH), severe OSA, etc., were compared using Pearson’s Chi-square tests, with Fisher’s exact test employed for cells with expected counts below five. Statistical significance was defined as a two-tailed *p*-value < 0.05. To control for the increased risk of Type I errors due to multiple comparisons, the Bonferroni correction was applied where appropriate.

Key predictor variables included OSA severity (categorical: mild/moderate/severe), subjective sleepiness (continuous: ESS score, range 0–24), insomnia severity (continuous: AIS score, range 0–24), and OSA risk (continuous: STOP-BANG score, range 0–8). Analyzed covariates comprised demographic factors (age, gender, etc.), objective physiological metrics (AHI, ODI, time spent with oxygen saturation < 90%, and nadir SpO_2_), and clinically relevant comorbidities, such as AH, Diabetes Mellitus (DM) and Coronary Artery Disease (CAD), all coded as binary (yes/no) variables.

Receiver operating characteristic (ROC) curve analysis was performed to evaluate the ability of three screening instruments—the STOP-BANG questionnaire, the ESS, and the AIS—to identify moderate-to-severe obstructive sleep apnea, defined as an apnea–hypopnea index (AHI) ≥ 15 events/hour. For each instrument, the Area Under the ROC Curve (AUC) was calculated as a measure of discriminatory power. Higher test scores were considered indicative of a positive disease state. All analyses were stratified by age group (18–45, 45–60, 60–75, and 75–90 years) and by sex (men, women, and the overall combined sample) to examine potential effect modification. ROC analyses were conducted using non-parametric methods, and asymptotic 95% confidence intervals for the AUC were requested. Due to the discrete nature of the screening scores, ties between positive and negative groups were present; the non-parametric ROC analysis remains valid under these conditions.

Missing data were managed using a multi-faceted approach. Patients with missing critical diagnostic or demographic variables—specifically AHI, age or sex—were excluded from the final analytic sample, as these were essential for primary classification and stratification (Figure 1). For patients with incomplete questionnaire data that precluded severity classification or risk stratification, complete-case analysis was applied, meaning these individuals were excluded only from analyses requiring those specific variables. However, they were retained in the overall sample for descriptive statistics where their available data could contribute.

For the remaining patients with partial missing data on individual questionnaire items or covariates (e.g., isolated missing pack-year data, occasional unanswered ESS or AIS questions), we performed available-case analysis, wherein all non-missing data were utilized for each specific analysis without imputation. Given the large sample size (*n* = 3736) and the relatively low proportion of missingness for most variables (<5% for key outcomes), listwise deletion was deemed acceptable for multivariable models, as it was unlikely to introduce substantial bias. No imputation techniques (e.g., multiple imputation, mean substitution) were employed, as our primary aim was to preserve the integrity of the original observed data, and the complete-case sample remained adequately powered for all planned analyses. Sample sizes for each variable are indicated in the table footnotes to maintain transparency.

## 3. Results

This study included 3736 patients diagnosed with OSA. The cohort was stratified into three age groups to examine the evolution of the disease across the lifespan: young adults (<45 years, *n* = 663), middle-aged adults (45–65 years, *n* = 1446), and older adults (>65 years, *n* = 1627). The sample was predominantly male (70.7%), reflecting the typical referral pattern to sleep clinics. Owing to resource limitations, most patients (N = 3228, 86.4%) underwent HSAT, while PSG (N = 508, 13.6%) was performed in patients with comorbidities (e.g., heart failure, pulmonary disease, or neuromuscular disorders) or unable to perform HSAT. The clinical and polysomnographic characteristics of the entire cohort, stratified by age, are presented in Table 1.

The severity of OSA, as measured by the AHI and ODI, differed significantly across age groups (*p* < 0.001 for both). Post hoc analyses revealed that both the 45–65 and >65 age groups had significantly higher AHI and ODI compared to the <45 group (all *p* < 0.001), with disease severity peaking in middle age. A clear, progressive worsening of nocturnal hypoxemia was observed with advancing age. The >65 group endured the greatest hypoxic index, spending significantly more time with SpO_2_ < 90% compared to both younger groups (*p* < 0.001).

The prevalence of cardiometabolic comorbidities also increased dramatically with age. Over half (59.7%) of patients >65 years had AH, compared to 33.7% in the 45–65 group and only 7.5% in the youngest group (*p* < 0.001); ORs (CI 95%) were 6.18 (4.54–8.41) for the 45–65 and 17.76 (13.16–23.96) for the >65 group. A similar steep increase was observed for DM and CAD (*p* < 0.001 for both). OR (CI95%) of DM for those between 45 and 65 was 3.23 (2.07–5.04) and for those over 65 was 9.58 (6.21–14.78), while regarding CAD, the respective values were 14.73 (4.66–46.57) and 47.94 (15.27–150.48). The cumulative smoking exposure, quantified as pack-years, also increased progressively across age groups, with older patients demonstrating significantly higher tobacco burden (*p* < 0.001). EDS, measured by the ESS, was most pronounced in the 45–65 group. A significant drop in ESS scores was observed in the >65 group despite high AHI values (*p* = 0.009), suggesting a shift towards a less symptomatic, hypoxic-non-sleepy phenotype in the elderly.

Gender-specific analysis of the entire cohort (N = 3736) revealed distinct clinical profiles (Table 2). Women were referred at a significantly older age (58.3 ± 12.0 vs. 56.5 ± 14.0 years, *p* = 0.003) and had a higher Body Mass Index (BMI) (38.0 ± 180.9 vs. 32.5 ± 9.5 kg/m^2^, *p* < 0.001) than men. Men had a higher AHI (34.3 ± 24.8 vs. 27.9 ± 23.3 events/h, *p* < 0.001) and a significantly higher smoking burden (26.9 ± 33 vs. 11.9 ± 19.5, *p* < 0.001), whereas women experienced a profoundly longer duration of nocturnal hypoxemia (Time SpO_2_ < 90%: 39.6 ± 314.2 vs. 21.7 ± 29.2 min, *p* = 0.001).

Symptom profiles were markedly different between genders. Women were significantly sleepier (ESS: 10.0 ± 4.7 vs. 9.2 ± 4.5, *p* < 0.001) and reported insomnia symptoms (AIS: 11.3 ± 5.2 vs. 7.3 ± 5.0, *p* < 0.001, Cohen’s d = −0.800). The clinical characteristics of female patients with OSA by menopausal status are presented in Table 3. Post-menopausal women (>45 years, *n* = 682) exhibited significantly higher AHI (36.8 ± 25.9 vs. 26.4 ± 22.1 events/h, *p* < 0.001, Cohen’s d = 0.43) and were 60% more likely to have severe OSA (49.6% vs. 31.3%, *p* < 0.001) compared to pre-menopausal women (<45 years, *n* = 412). ODI and Time SpO_2_ < 90% were greater in the post-menopausal group.

The burden of cardiometabolic comorbidities nearly doubled or tripled after menopause (Table 3). The odds of having AH and CAD were 3 and 4 times higher, respectively, in post-menopausal women (*p* < 0.001 for all). While sleepiness remained constant, the severity of insomnia was significantly worse in the older group (*p* < 0.001).

The predictive performance of the ESS, STOP-BANG, and AIS for detecting moderate-to-severe OSA (AHI ≥ 15) was evaluated across age and gender subgroups (Table 4). This refined stratification was employed to detect clinically significant variations in test accuracy that may be masked by broader categories, particularly in older populations. The STOP-BANG questionnaire was the most robust tool overall, consistently achieving AUC values closest to 0.7 across all demographics.

The predictive Performance was superior in men compared to women, particularly in the youngest groups. For women under 45 years, the predictive value of the ESS (AUC = 0.447) and AIS (AUC = 0.409) was particularly poor. A notable shift was observed in the oldest-old group (75–90 years), where traditional tools like STOP-BANG performed worse. Intriguingly, in older women, the ESS and AIS demonstrated the highest predictive value (AUC = 0.684 and 0.676, respectively), contrary to the overall trend.

In multivariable analyses, ESS showed limited independent associations across age groups, with no significant predictors in patients <45 or 60–75 years, a weak association with BMI in the 45–60 years group, and an association with male sex in the 75–90 years group. In contrast, the STOP-BANG questionnaire demonstrated consistent independent associations across all age strata, mainly with BMI and AH, and additionally with male sex in younger patients. AIS showed age-dependent associations, including links with BMI, cardiometabolic comorbidities, AHI, and female sex, but no consistent pattern across all groups. Overall, STOP-BANG remained the most stable predictor after adjustment. In more detail for the ESS, multivariable analysis showed no statistically significant associations in the <45 years and 60–75 years age groups. In the 45–60 years group, ESS score was significantly associated with BMI (OR 1.018, 95% CI 1.002–1.034, *p* = 0.026). In the 75–90 years group, a significant association was observed with male sex (OR 1.95, 95% CI 1.053–3.615, *p* = 0.033). For the STOP-BANG questionnaire, multivariable analysis demonstrated significant associations across all age groups. In the <45 years group, STOP-BANG score was significantly associated with BMI (OR 1.44, 95% CI 1.329–1.56, *p* < 0.001), AH (OR 18.28, 95% CI 4.26–37.4, *p* < 0.001), and male sex (OR 6.28, 95% CI 2.26–17.4, *p* < 0.001). In the 45–60 years group, significant associations were observed with BMI (OR 1.23, 95% CI 1.169–1.297, *p* < 0.001) and arterial hypertension (OR 16.6, 95% CI 7.86–35.1, *p* < 0.001). In the 60–75 years group, STOP-BANG score remained significantly associated with BMI (OR 1.154, 95% CI 1.09–1.22, *p* < 0.001) and AH (OR 12.6, 95% CI 6.79–23.3, *p* < 0.001). In the 75–90 years group, significant associations were found for BMI (OR 1.325, 95% CI 1.144–1.53, *p* < 0.001) and AH (OR 5.18, 95% CI 1.7–15.75, *p* = 0.04). For the AIS, multivariable analysis showed no significant associations in the <45 years group. In the 45–60 years group, significant associations were found with BMI (OR 1.02, 95% CI 1.00–1.04, *p* = 0.048), AH (OR 1.50, 95% CI 1.114–2.029, *p* = 0.008), Diabetes Mellitus (OR 1.347, 95% CI 1.01–1.797, *p* = 0.04), and female sex (OR 0.54, 95% CI 0.39–0.736, *p* < 0.001). In the 60–75 years group, AIS score was significantly associated with AHI (OR 1.03, 95% CI 1.006–1.049, *p* = 0.011) and female sex (OR 0.54, 95% CI 0.39–0.736, *p* < 0.001). In the 75–90 years group, the only significant association was observed for female sex (OR 0.41, 95% CI 0.185–0.914, *p* = 0.029).

## 4. Discussion

The “typical” OSA patient is not one entity. The disease manifests differently across the lifespan and between genders. Screening and treatment approaches must account for these differences, particularly the under-recognition of OSA in women who may present with insomnia and fatigue rather than classic snoring and witnessed apneas. The findings of this study highlight crucial clinical manifestations of OSA depending on sex and age.

OSA severity, as measured by the AHI and ODI, is significantly higher in both the 45–65 and >65 age groups compared to the youngest group (<45). It peaks in the 45–65 cohort and remains severely elevated in the elderly. This ‘peak and plateau’ model identifies mid-life as a pivotal period where pathophysiological processes, such as hormonal changes and cumulative anatomical alterations, converge to maximize disease severity, while the persistence of severe OSA in the elderly marks its prevalence in older age and the urge for therapeutic interventions. These findings highlight the potential importance of treatment intervention, as exposure to severe OSA from mid-life may contribute to accelerated cognitive and cardiovascular risk [14].

Furthermore, the >65 group endures the greatest hypoxic indices (ODI, Time with SpO_2_ < 90%). This clear, progressive worsening of nocturnal hypoxemia with advancing age carries critical implications for both the pathophysiology and clinical management of OSA in the elderly population. Elderly patients often have multiple comorbidities, including AH, Chronic Obstructive Pulmonary Disease (COPD), Heart Failure (HF), cancer, and dementia [17]. Reduced respiratory reserve, blunted chemoreflex responses, and an increased prevalence of comorbidities, such as HF and COPD, probably act synergistically with OSA, leading to impaired oxygenation and augmented hypoxic stress [18]. This finding highlights substantial risk in older patients, many of whom may show only a moderate AHI on PSG. Given that nocturnal hypoxia can aggravate organ damage, neurocognitive decline, and cardio-pulmonary disease, early detection and proactive treatment warrant consideration. The marked age-related increase in hypoxic burden and cardiometabolic comorbidity might also be linked to the upregulation of inflammatory pathways. C-reactive protein (CRP), fibrinogen, and the Systemic Immune-Inflammation Index (SII) have been found to not only correlate with OSA severity but also track treatment response [19]. Since intermittent hypoxia is a potent driver of oxidative stress and pro-inflammatory cascades [18], elevated inflammatory markers such as CRP and fibrinogen in severe OSA could represent both a consequence of nightly hypoxic events and a mediator of end-organ injury, contributing to the high prevalence of AH, DM, and CAD in our older cohort.

Consistent with this, our study shows a marked age-related rise in cardiometabolic comorbidity: >50% of patients >65 years old had AH, while both DM and CAD increased significantly with age. However, it has been found that young adults with probable OSA present higher prevalence rates of any cardiovascular events, AH and metabolic syndrome [20,21]. This is still a topic of debate; a large retrospective study in the United States showed that the risk of hospitalization in older patients (>65 years) with cardiovascular disease was doubled in the year prior to an OSA diagnosis compared to non-OSA patients, suggesting that the impact of OSA remains significant in this age group [22]. Nevertheless, cardiovascular and metabolic comorbidities increase in OSA patients with aging, impacting clinical outcomes and Positive Airway Pressure (PAP) adherence, since PAP therapy might reduce the risk of cerebrovascular and cardiovascular events by lowering intermittent hypoxia and autonomic dysfunction [23].

Regarding sex differences, women present with OSA at an older age and higher BMI than men despite a lower AHI, suggesting systematic diagnostic delay driven by a male-centric OSA stereotype and women’s more “non-classical” complaints (insomnia, fatigue) being misattributed to depression or perimenopause [24,25]. In those <55 years, women more often report anxiety, insomnia, fatigue, and headaches, with depression and anxiety as key comorbidities [26], supporting the need for higher clinical suspicion for OSA in women with elevated BMI and atypical sleep complaints. Men may exhibit a more anatomy-driven phenotype, potentially influenced by longer smoking exposure that promotes airway inflammation and neuromuscular impairment, worsening collapsibility [27], alongside a higher burden of CAD, AH, Atrial Fibrillation (AF), and other cardiac diseases [26]. In contrast, women show a critical discordance: lower AHI yet longer nocturnal hypoxemia, indicating that AHI alone may underestimate severity and cardiovascular risk. Mechanistically, reduced oxygen-carrying capacity and increased oxygen demand, plus loss of progesterone-related ventilatory protection with age, may amplify sustained hypoxemia [11,28]. Therefore, hypoxia metrics should be routinely reported and integrated into risk stratification, consistent with the modified Baveno classification [29].

Women also report greater sleepiness and markedly more insomnia (higher ESS and AIS), implying a phenotype in which insomnia may be a core feature, potentially related to heightened arousal and mood comorbidity [24,30,31]. Clinically, OSA should be actively considered in women presenting with insomnia and/or fatigue, and management should target hypoxic burden and insomnia alongside AHI reduction. Menopausal status emerges as a major inflection point for OSA severity in women. Post-menopause aged women show higher AHI (36.8 vs. 26.4) and more severe OSA (49.6% vs. 31.3%), supporting a protective role of pre-menopausal sex hormones [11]. They also exhibit longer hypoxemia (80% more time with SpO_2_ < 90%) and a markedly higher cardiometabolic comorbidity load, consistent with synergy between menopause-related metabolic changes (visceral adiposity, insulin resistance) and OSA-induced hypoxemia, accelerating cardiovascular and metabolic disease [28,32]. Clinically, our findings suggest that menopause may represent an inflection point for OSA severity, supporting consideration of sleep testing in post-menopausal women with even mild symptoms, with attention to hypoxemia and cardiovascular risk factors [33].

Menopausal status appeared to coincide with a shift toward a more severe and hypoxic OSA phenotype in women, although this finding should be interpreted with caution because menopausal status was approximated using age rather than confirmed clinical history or hormonal measurements. Women older than 45 years showed higher AHI values, longer nocturnal hypoxemia, and a greater burden of cardiometabolic comorbidities than younger women. These findings align with previous reports suggesting that hormonal changes, redistribution of body fat, and age-related changes in ventilatory control may worsen sleep-disordered breathing in midlife and older women. However, given the cross-sectional design and use of age as a proxy for menopause, our results should be considered hypothesis-generating rather than evidence of a direct menopausal effect. An 80% increase in time spent with SpO_2_ < 90% indicates substantially greater hypoxic strain; thus, for a similar AHI, women over 45 years may sustain greater end-organ injury [28]. Management could therefore prioritize hypoxemia reduction (not AHI alone) and adopt a lower threshold for evaluating hypoxia-related complications such as pulmonary hypertension and arrhythmias [33]. In parallel, cardiometabolic comorbidities nearly double or triple after age 45, consistent with a synergistic interaction between menopause-related metabolic changes (visceral adiposity, insulin resistance) and hypoxic OSA that accelerates cardiovascular risk [32]. The increase in hypoxic burden and comorbidity prevalence in women over 45 may reflect the combined influence of aging, metabolic changes, and loss of hormonal protection rather than menopause alone. Thus, this transition period may be a clinically important stage for increased vigilance and earlier diagnostic evaluation. Further prospective studies with precise characterization of menopausal status are needed to confirm the mechanisms behind these differences.

Symptomatically, older women increasingly fit a Comorbid Insomnia and Sleep Apnea (COMISA) phenotype: insomnia severity rises with age while ESS may remain unchanged, making “classic” sleepiness-based screening unreliable [34]. Clinically, PAP alone may not resolve the sleep complaint unless insomnia is treated concurrently, supporting integrated management. Emerging adjuncts include atomoxetine–oxybutynin to reduce OSA severity [35] and Glucagon-Like Peptide-1 (GLP-1) agonists, which improve AHI and may also benefit insomnia indirectly via metabolic and respiratory improvements [36]. Overall, menopause transition should be considered a major risk factor for OSA. Clinically, women in the post-reproductive years constitute a distinct high-risk group, warranting a lower threshold for screening and a more intensive, phenotype-informed approach that SDB and sleep hygiene—particularly when symptoms are atypical (e.g., insomnia) and cardiometabolic comorbidities are present. These findings also justify further investigation into whether hormone replacement therapy could mitigate OSA severity in selected, recently post-menopausal women [37]. However, given the limitations of age-based proxy classification, any potential role for hormone replacement therapy remains speculative and requires rigorous evaluation in studies with confirmed menopausal status and hormonal profiling.

Our study highlighted differences in the predictive values of different OSA screening tools across genders and age groups. The ESS dropped significantly in those >65 despite high AHI, suggesting a “hypoxic–non sleepy” elderly phenotype in contrast to younger, more sleepy patients [38]. Reduced arousability, competing sleep fragmentation (e.g., insomnia), and age-related neurobiological changes may blunt perceived sleepiness, making ESS less reliable in older adults [39,40,41]. This may leave clinically significant OSA underdiagnosed and shift risk toward hypoxia-driven outcomes (cardiometabolic disease, cognitive decline), suggesting assessment beyond ESS using indices like ODI plus cardiometabolic screening [42].

STOP-BANG demonstrated moderate discriminatory ability (AUC ~ 0.7) across most subgroups, supporting its utility as a practical first-line screening tool in diverse populations. However, the age- and sex-specific variations in performance highlight the need for cautious interpretation and potential adjustment of thresholds in certain subgroups [43]. The decline for moderate–severe OSA discrimination in those >65 years likely reflects age-driven score inflation (neck circumference, AH, BMI), producing a ceiling effect that reduces specificity in older, multimorbid cohorts and risks over-referral [44]. The performance drop in elderly women also aligns with a phenotype shift: less “classic” presentation, reduced observed apneas (living alone), and symptoms such as sleepiness masked by comorbidities or insomnia [6,26]. Furthermore, Aliyeva and colleagues’ application of machine learning to identify CRP, SII, and fibrinogen as top predictors of severity [19] underscores the critical gap that anatomical factors-oriented screening tools like STOP-BANG fail to address. Despite being robust first-line screeners, they do not capture the full biological variability of the disease and inflammation-related risk in different demographics. Collectively, these findings argue against a one-size-fits-all interpretation and support age- and sex-adjusted STOP-BANG thresholds (e.g., decade-specific cut-offs), particularly for geriatric women.

Screening tools performed better in men than women, with the largest gap in the <45 cohort: in young women, ESS and AIS showed near-random discrimination (AUC ~ 0.45). This indicates that symptom-based screening in pre-menopausal women is not merely weak but potentially misleading, systematically missing OSA and reinforcing under-diagnosis. Clinicians should therefore treat fatigue, insomnia, and mood complaints in young women as potential OSA signals rather than excluding OSA on the basis of low “classic” sleepiness scores [24]. This disparity also supports a sex-specific pathophysiology in pre-menopausal women, where OSA may be less “sleepiness-dominant” and more influenced by non-anatomical traits not captured by ESS and/or AIS. A plausible mechanism is a lower arousal threshold in women: mild flow limitation or increased airway resistance may trigger earlier cortical arousal before substantial desaturation, producing fragmented sleep without prominent hypoxemia or marked ESS elevation [30].

Among older women, a screening paradox emerges. STOP-BANG scores are often high—largely due to age-related increases in hypertension and BMI [12]—yet the clinical presentation is frequently non-classical, dominated by insomnia and “tiredness” rather than loud snoring or witnessed apneas. Thus, while STOP-BANG captures physiological risk in post-menopausal women, its discrimination may be blunted by score inflation and by female-specific symptom expression.

Conversely, ESS performs relatively best in older women (AUC 0.684). This likely means that when a woman >75 reports clear, significant EDS, it is a highly specific red flag for clinically relevant OSA in this age stratum—especially because sleepiness is often minimized, reframed as fatigue, or attributed to aging, comorbidity, or polypharmacy [30]. In practice, ESS shifts from a weak population screener to a useful “rule-in” signal in very old women.

Overall, these findings reflect a bipolar screening approach in women: in younger patients, objective risk factors (BMI, comorbidities, and STOP-BANG) may carry greater weight than symptom scores, whereas in older women, prominent ESS-defined sleepiness may be an important clinical indicator of significant OSA. In addition, after multivariable adjustment, STOP-BANG showed consistent independent associations across all age groups, mainly with BMI and arterial hypertension, indicating stable performance across the lifespan. In contrast, ESS and AIS demonstrated weaker and age-dependent associations, with limited or inconsistent predictors after adjustment, particularly in younger and older patients.

As part of our research future directions, large, population-based cohorts are essential to confirm the generalizability of our sex- and age-specific findings beyond sleep clinic settings. Longitudinal, prospective cohort studies are needed to map OSA trajectories across key life stages (e.g., menopause, aging) and establish causality, moving beyond cross-sectional associations. Regarding refinement of screening tools, age- and sex-adjusted STOP-BANG thresholds are required to improve accuracy in older adults, as well as developing new screening instruments for pre-menopausal women that capture fatigue, insomnia, and mood disturbance apart from classic sleepiness. Moreover, hypoxic metrics (ODI, time SpO_2_ < 90%) and inflammatory phenotyping need to be incorporated into risk stratification, given their discordance with AHI in female and elderly patients. Geriatric-specific diagnostic criteria warrant investigation, particularly whether treatment decisions should prioritize hypoxic burden over AHI to prevent cognitive decline and cardiovascular events in non-sleepy older adults. Additionally, interventional trials should investigate hormone replacement therapy as an adjunct treatment in recently post-menopausal women, while Cognitive Behavioral Therapy for Insomnia (CBT-I) in older women could be evaluated more thoroughly as an adjuvant intervention to COMISA management. These directions will support the transition from descriptive phenotyping to personalized, life-course approaches in OSA management.

## 5. Limitations

Several methodological limitations should be considered. First, the cross-sectional, clinic-based design identifies associations but cannot establish causality regarding aging- or menopause-related changes in OSA phenotype. Symptom burden (ESS, AIS) and smoking history were self-reported, introducing recall and reporting bias. Regarding missing data, we employed complete-case and available-case analyses without imputation. While this approach preserves the integrity of observed responses and is appropriate given the large sample size and low missingness rate (<5% for most variables), it may introduce bias if data were not missing completely at random. However, sensitivity analyses comparing included and excluded patients on available demographic variables revealed no significant differences in age, sex distribution, or OSA severity, suggesting that exclusion bias is likely minimal. The single-center, male-predominant referral cohort also creates selection bias, likely overrepresenting the “classic” male phenotype and underrepresenting milder or atypical community cases—particularly women—thereby limiting external validity of sex-specific conclusions. Our study included sleep studies performed with both PSG and HSAT, which may introduce methodological heterogeneity, as diagnostic modality can influence AHI estimation, hypoxemia indices, and screening questionnaire performance. The number of PSG recordings was relatively small compared with HSAT, reflecting real-world practice, and therefore, a PSG-only sensitivity analysis was not feasible. Although diagnostic modality was included as a covariate in multivariable analyses, residual confounding cannot be fully excluded.

Menopausal status was approximated using age (≤45 vs. >45 years). Although pragmatic, this approach lacks the precision of clinical menopause history or hormonal assays (not consistently available), potentially misclassifying perimenopausal or surgically menopausal women. Residual confounding is also possible because medication use, hormonal variability, and socioeconomic factors were not captured. We used different age strata for descriptive analyses (three groups) versus questionnaire validation (four groups); this was intentional to provide decade-specific, clinically actionable performance estimates, supported by the large overall sample, though subgroup estimates may still be less stable.

Finally, screening tools showed only modest discrimination (AUC < 0.8) across subgroups, implying that unmeasured physiological traits and contextual factors explain substantial residual variance. The very low AUCs for symptom-based tools in young women may also reflect their underrepresentation in clinic cohorts, where attendees may constitute a more severe or atypical subset. Overall, findings are most generalizable to sleep-clinic populations. Future work should use prospective, population-based designs with precise menopause characterization and multidimensional physiological phenotyping to clarify causal pathways across the lifespan in both sexes.

## 6. Conclusions

In conclusion, this study demonstrates that OSA is not a singular disorder but a condition characterized by dynamic, demographic-specific phenotypes. The ineffectiveness of symptom-based tools in young women, along with the altered predictive value of standardized questionnaires in older adults, underscores the urgent need for a personalized, life-course approach to OSA. Gender differences arise from a complex interplay of biological factors—most notably illustrated by the post-reproductive years transition—and clinical biases that lead to delays in diagnosis and an underestimation of hypoxic risk in women. Future clinical practice should incorporate these findings by adopting sex- and age-stratified screening strategies and by redefining disease severity to include hypoxic burden. This approach will help ensure timely diagnosis and personalized management for all patients throughout their lifespan.

## Figures and Tables

**Table 1:** Clinical and polysomnographic characteristics of patients with obstructive sleep apnea by age group.

Variable	<45 Years (*n* = 663)	45–65 Years (*n* = 1446)	>65 Years (*n* = 1627)	*p*-Value
Male Sex, n (%)	550 (83.0%)	1015 (70.2%)	1081 (66.4%)	<0.001
Body Mass Index (BMI), kg/m^2^	30.99 ± 8.61	32.86 ± 9.24	32.14 ± 9.06	<0.001
Pack-Years	10.13 ± 13.85	22.15 ± 25.60	28.51 ± 38.23	<0.001
Epworth Sleepiness Scale (ESS)	9.29 ± 4.57	9.91 ± 4.72	9.49 ± 4.45	0.009
STOP-BANG Score	3.60 ± 1.59	4.83 ± 1.74	5.22 ± 1.67	<0.001
Apnea Hypopnea Index (AHI), events/h	25.68 ± 27.42	35.00 ± 25.93	33.36 ± 21.73	<0.001
Oxygen Desaturation Index (ODI), events/h	24.87 ± 26.79	34.56 ± 26.18	33.36 ± 22.59	<0.001
Average SpO_2_, %	93.37 ± 3.05	92.07 ± 3.20	91.20 ± 3.97	<0.001
Time with SpO_2_ < 90%, min	13.58 ± 23.92	20.29 ± 28.07	28.51 ± 33.46	<0.001
Arterial Hypertension (AH), n (%)	50 (7.5%)	488 (33.7%)	972 (59.7%)	<0.001
Diabetes Mellitus (DM), n (%)	23 (3.5%)	151 (10.4%)	417 (25.6%)	<0.001
Coronary Heart Disease (CAD), n (%)	3 (0.5%)	92 (6.4%)	296 (18.2%)	<0.001

*Data are presented as mean ± standard deviation or n (%). Sample sizes vary per variable due to missing data; column headers indicate the maximum available n for each age group. p-values were derived from one-way ANOVA for continuous variables and Pearson’s χ^2^ test for categorical variables.*

**Table 2:** Gender-specific differences in the sleep clinic cohort.

Variable	Males (N = 2.642)	Females (N = 1.094)	*p*-Value
Age, years	56.5 ± 14	58.34 ± 12	0.003
AHI, events/h	34.3 ± 24.8	27.9 ± 23.3	<0.001
BMI, kg/m^2^	32.5 ± 9.5	38 ± 18.9	<0.001
Pack-years	26.9 ± 33	11.9 ± 19.5	<0.001
Time Sat < 90%	21.7 ± 29.2	39.6 ± 314.2	0.001
ODI, events/h	32.4 ± 24.3	29.3 ± 25.3	<0.001
ESS score	9.2 ± 4.5	10 ± 4.7	<0.001
STOP BANG score	4.6 ± 1.8	5 ± 1.8	<0.001
AIS score	7.3 ± 5	11.3 ± 5.2	<0.001
Severe Obstructive Sleep Apnea (OSA) %	46.5	33.1	<0.001

*Data are presented as mean ± standard deviation or percentage (%). p-values were derived from independent samples t-tests (continuous variables) and Pearson’s χ^2^ tests (categorical variables). Sample sizes reflect the number of patients with complete data for each variable; totals are indicated in column headers.*

**Table 3:** Clinical characteristics of female patients with OSA stratified by age (≤45 vs. >45 as a proxy for menopausal status).

Characteristic	<45 Years (Pre-Menopausal) (*n* = 412)	>45 Years (Post-Menopausal) (*n* = 682)	*p*-Value	Effect Size
BMI, kg/m^2^	34.2 ± 8.1	38.6 ± 9.3	<0.001	Cohen’s d = 0.51
AHI, events/h	26.4 ± 22.1	36.8 ± 25.9	<0.001	Cohen’s d = 0.43
ODI, events/h	25.1 ± 21.8	35.2 ± 25.3	<0.001	Cohen’s d = 0.43
Time SpO_2_ < 90%, min	18.2 ± 26.4	32.7 ± 35.1	<0.001	Cohen’s d = 0.46
ESS score	9.8 ± 4.5	10.1 ± 4.8	0.320	Cohen’s d = 0.06
AIS score	10.4 ± 5.0	11.8 ± 5.3	<0.001	Cohen’s d = 0.27
Severe OSA, %	31.3%	49.6%	<0.001	OR = 2.16 (1.64–2.85)
AH, %	32.1%	58.7%	<0.001	OR = 3.00 (2.3–3.92)
DM, %	8.3%	19.2%	<0.001	OR = 2.62 (1.77–3.89)
CAD, %	3.2%	11.4%	<0.001	OR = 3.89 (2.26–7.01)

*Menopausal status was inferred using an age cut-off of 45 years. This proxy does not account for individual variation in menopausal onset, perimenopause, surgical menopause, or hormone therapy, and findings should be interpreted with this limitation in mind. Data are presented as mean ± SD or % (n). p-values were calculated using independent samples t-tests (continuous variables) or Pearson’s χ^2^ tests (categorical variables). Effect sizes are reported as Cohen’s d for continuous measures and as odds ratios (ORs) with 95% confidence intervals for binary outcomes.*

**Table 4:** Predictive performance (AUC) of screening tools for AHI ≥ 15.

Group	STOP BANG (95% CI)	ESS (95% CI)	AIS (95% CI)
**Total**			
**<45 yrs**	0.747 (0.699–0.795)	0.614 (0.559–0.670)	0.557 (0.500–0.614)
**45–60 yrs**	0.740 (0.703–0.777)	0.607 (0.564–0.650)	0.539 (0.494–0.585)
**60–75 yrs**	0.744 (0.700–0.788)	0.620 (0.572–0.668)	0.566 (0.514–0.618)
**75–90 yrs**	0.696 (0.605–0.786)	0.645 (0.552–0.738)	0.540 (0.439–0.642)
**Men**			
**<45 yrs**	0.746 (0.693–0.798)	0.644 (0.585–0.704)	0.586 (0.525–0.647)
**45–60 yrs**	0.751 (0.706–0.797)	0.626 (0.571–0.680)	0.595 (0.537–0.653)
**60–75 yrs**	0.747 (0.691–0.803)	0.610 (0.547–0.673)	0.579 (0.513–0.646)
**75–90 yrs**	0.694 (0.581–0.808)	0.624 (0.498–0.751)	0.478 (0.353–0.603)
**Women**			
**<45 yrs**	0.731 (0.600–0.863)	0.447 (0.296–0.598)	0.409 (0.258–0.560)
**45–60 yrs**	0.718 (0.653–0.784)	0.576 (0.503–0.649)	0.515 (0.440–0.589)
**60–75 yrs**	0.743 (0.673–0.813)	0.630 (0.553–0.706)	0.576 (0.492–0.661)
**75–90 yrs**	0.703 (0.549–0.857)	0.684 (0.537–0.830)	0.676 (0.502–0.850)

*AUC values and 95% confidence intervals were derived from non-parametric ROC analysis using the DeLong method. Standard errors are available from the authors upon request. The reference standard was an AHI ≥ 15 on PSG or HSAT. Analyses were performed separately for each age stratum and for the total cohort, men, and women.*

## Data Availability

The raw data supporting the conclusions of this article will be made available by the authors on request.
